# CLU: A new algorithm for EST clustering

**DOI:** 10.1186/1471-2105-6-S2-S3

**Published:** 2005-07-15

**Authors:** Andrey Ptitsyn, Winston Hide

**Affiliations:** 1 Pennington Biomedical Research Center, 6400 Perkins Rd. Baton Rouge LA 70808; 2 South African National Bioinformatics Institute, P/b X17 UWC SANBI Bellville 7535

## Abstract

**Background:**

The continuous flow of EST data remains one of the richest sources for discoveries in modern biology. The first step in EST data mining is usually associated with EST clustering, the process of grouping of original fragments according to their annotation, similarity to known genomic DNA or each other. Clustered EST data, accumulated in databases such as UniGene, STACK and TIGR Gene Indices have proven to be crucial in research areas from gene discovery to regulation of gene expression.

**Results:**

We have developed a new nucleotide sequence matching algorithm and its implementation for clustering EST sequences. The program is based on the original CLU match detection algorithm, which has improved performance over the widely used d2_cluster. The CLU algorithm automatically ignores low-complexity regions like poly-tracts and short tandem repeats.

**Conclusion:**

CLU represents a new generation of EST clustering algorithm with improved performance over current approaches. An early implementation can be applied in small and medium-size projects. The CLU program is available on an open source basis free of charge. It can be downloaded from

## Background

Expressed sequence tags (ESTs) represent a significant advancement in modern biology. With their introduction in early 90's they represent the first truly high-throughput technology that deluged the databases and made the advent of advanced computer technologies in biology inevitable. The flood of these short, error-prone messages represents another important, although not immediately obvious revolution: it has heralded the transition of modern biology from genetics to the genomics era. ESTs have offered the first glimpse at the transcriptome, i.e. a volume of messages, copied from genes and forwarded to all corners of a living cell. An EST library is essentially a coarse-grained snapshot of all mRNA molecules present at a given time in a biological sample. Currently there are more accurate and advanced technologies to analyze the function of genomes, but EST sequencing was one of the first approaches and is still in extensive use today.

**Figure 1:**
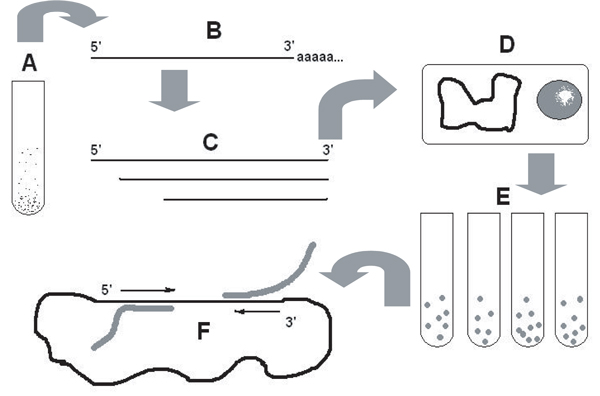
Schematic outline of a typical EST manufacture process. After a sample of interest (A) is collected all transcribed copies of expressed genes (mRNAs) are isolated (B). Each mRNA is reverse-transcribed into a complementary DNA (cDNA) (C). Note that cDNA copies may have different lengths due to the polymerase processivity. The copy number of those fragments is increased when they are inserted into a host cell (D). The resulting population of host cells, containing cDNA fragments from the sample of interest is called a cDNA library (E). Typically a few thousand clones are randomly picked from the cDNA library to produce single-pass reads from 3', more rarely from the 5' ends and sometimes from the random location in the middle (F). The resulting collection represents fragments of different lengths starting from the polyadenylation site at 3' end and partially overlapping fragments from 5' end, starting at different points. Although 3' and 5' ESTs rarely overlap, they usually share the same clone ID in the annotation.

For EST sequences, only a few hundred readable bases are produced from each sequencing read, and yet a full gene transcript may be several thousands of bases long (Figure 1 outlines the EST production process). In publicly available databases, EST length varies from less than 20 to over 7000 base pairs, with an average length of 360 base pairs and standard deviation of 120 base pairs (data from dbEST, Genbank rel. 104). Obviously, not all of these sequences are true single-read tags, but they are submitted and accepted as such, bringing extra complications to EST analysis. There is significant diversity in EST generation methods. One of the most significant is using random primers, which results in production of fragments without direction, originating from different non-overlapping parts of the same mRNA [1]. ESTs provide a "tag level" association with an expressed gene sequence, trading quality and total sequence length for the high quantity of samples.

A large scale and systematic public effort to isolate all human genes began in 1993 when the Integrated Molecular Analysis of Genomes and their Expression (IMAGE) consortium was formed to create, collect and characterize cDNA libraries from various tissues and different state of normalization [2]. This initiative gained significant momentum when Merck & Co. provided funding to the Washington University Genome Sequencing Center to partially sequence clones from the IMAGE cDNA libraries to generate expressed sequence tags. EST sequences are now submitted to dbEST – a special division of Genbank [3]. Apart from the centralized resources there are countless smaller databases, scattered throughout academic and commercial research laboratories, often available for download. An example of such ongoing EST sequencing project Rat EST can be found at the University of Iowa . Large quantities of ESTs are also collected among other data in disease-oriented resource centers such as Cancer Genome Anatomy Project at the National Cancer Institute, USA .

Practical application of a particular EST clustering technique results in a database of clustered ESTs, often including mRNA, genomic DNA sequence and other available information: a so called gene index. The primary goal of most gene indices is to reconstruct the gene complement of a genome and therefore require strict criteria to assign one gene to one cluster. This approach is implemented by TIGR [4], UniGene [5], IMAGEne [6] and MERCK [7]. A novel approach, implemented by STACK [8] aims to capture transcript variation in the context of developmental and pathological states. This approach incorporated additional steps that were tolerant of sub-sequence diversity and the ability to perform assembly analysis. The gene indexing projects described below implement a combination of data preparation, clustering, assembly, alignment analysis, consensus generation, clone linking and visualization.

In order to achieve maximum performance with minimal compromise on sensitivity we have developed a new algorithm for nucleic acid sequence comparison. Recently, the growth rate of sequence data in genomic databases has surpassed the growth rate of computer performance, mostly due to introduction of high throughput sequencing technologies like EST manufacture and to a multiplicity of genome sequencing projects. So far the existing clustered EST databases manage to cope with the deluge of data, but only with a help of high-end computer facilities. Projection of this situation into the future suggests that the progress of computer hardware design may not be sufficient. New algorithmic approaches are required to mitigate this problem.

Some latest developments in the EST clustering systems give preference to fast sub-linear algorithms [9]. The sheer speed of sequence comparison is the obvious advantage of this family of algorithms. There are also some disadvantages. First of all, the speed comes at the price of precision. A higher false-positive rate is compensated by a thorough alignment at the subsequent cluster assembly stage. False-negative cases, once dropped, are hard to detect. To cut the probable loss of matching sequence pairs, clustering programs have to be tuned to higher sensitivity. As a result, the slow cluster assembly stage becomes overloaded with sequences that can't be aligned (i.e. false-positives). Many fast sub-linear algorithms require an extensive pre-clustering procedure. Apart from being resource consuming, this stage is also a difficult parallel optimization. Pre-calculated index tables need to be adjusted with each update of the initial data set. A clustering system, built around a fast, but imprecise sub-linear algorithm would require lots of compensatory mechanisms and additional routines. Complex structure obstructs further development of the clustering system as a whole.

In spite of the seemingly obvious choice of the fastest possible algorithm we experiment here with another strategy. Although linear class algorithms are generally slower then sub-linear, they have some advantages as a basis of an EST clustering system. A more precise algorithm would produce much less false-positive and false-negative results. Sensitivity to small regions of local similarity can improve quality of results by detecting short, but non-random overlaps between EST fragments and may result in much longer consensus sequences. Detecting small regions of similarity, even if accompanied by much longer non-similar stretches, is crucial for detecting alternative gene variants.

## Methods

### Fast algorithm for sequence comparison

Suppose we have a query nucleic acid sequence (EST, for example) *S*_*q*_. We are to compare this query sequence to a sequence *S*_*b *_to reveal their possible similarity. To consider only words situated within a short distance from each other we introduce a short frame sliding by one position at a time along *S*_*b*_. We compare the words found anywhere in the sequence *S*_*q *_to the words found in each frame of *S*_*b*_. The frame containing few or none of coinciding words is most probably unrelated to *S*_*q*_. A high number of words, coincident between *S*_*q *_and a short frame in *S*_*b*_, may indicate a zone of local similarity, providing that the words are informative (not too abundant). To accelerate the comparison all words found in sequence *S*_*q *_are presented in a table (*H*). The table *H *is a linear array, where the offset itself is a hash value of certain oligonucleotide. Each element of this array contains a number, associated with a corresponding oligonucleotide. Each value of the hash table *H *can be differentially weighted. Some words, for example repeats of one letter, are less informative. Words over-abundant in a sequence *S*_*q *_are likely to be less informative than unique or rare words. Differential weighting allows ignoring low-complexity regions and short tandem repeats without extensive pre-processing.

We slide the frame *W *repeatedly along S_*b *_by one letter at a time, calculating a similarity function *F(W*_*i*_*) *for each frame *W*_*i*_.



Where *w *is the width (number of words) of the frame *W*.

**Figure 3:**
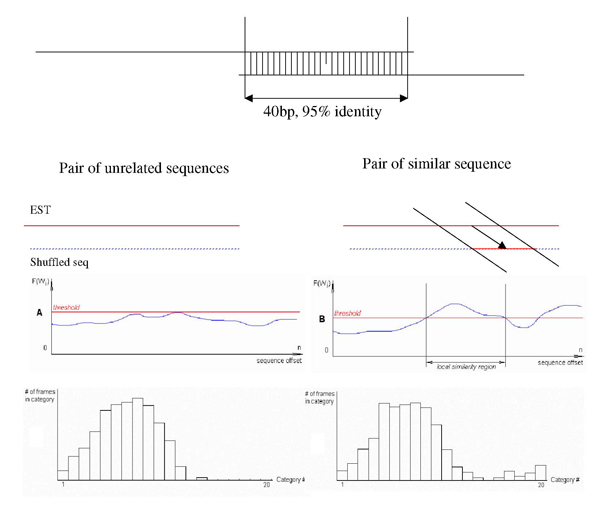
Generation of simulated non-matching (A) and matching (B) pairs in the process of algorithm development. A non-matching pair of sequences is generated by shuffling a randomly picked EST. A matching pair is generated by coping a stretch of 40 base pairs with one mismatch from one sequence to another. For each pair the local similarity function F(Wi) profile and categorized distribution of its values is generated.

The similarity function *F(W*_*i*_*) *forms a continuous profile of local similarity of each frame of *S*_*b *_to the query sequence *S*_*q*_. If two unrelated sequences are compared, there will be still non-zero values if *F(W*_*i*_*) *due to the randomly occurring matching words. If two sequences contain even a short area of strong similarity, this area will be reflected as a surge of *F(W*_*i*_*) *like it is presented on Figure 3. A heuristic algorithm for scanning sequence databases for matching sequences based on this idea has been developed in early 90 s [10]. However that algorithm did not provide quantitative estimation of sequence similarity or reliability of a match. To derive a single similarity score for a pair of sequences we transform the similarity function *F(W*_*i*_*) *values to a categorized distribution form taking the number of base pairs within a frame *W *for number of categories. The observed distributions for the pairs of unrelated sequences are different from the distributions, derived from related sequences (see Figure 3).

**Figure 4:**
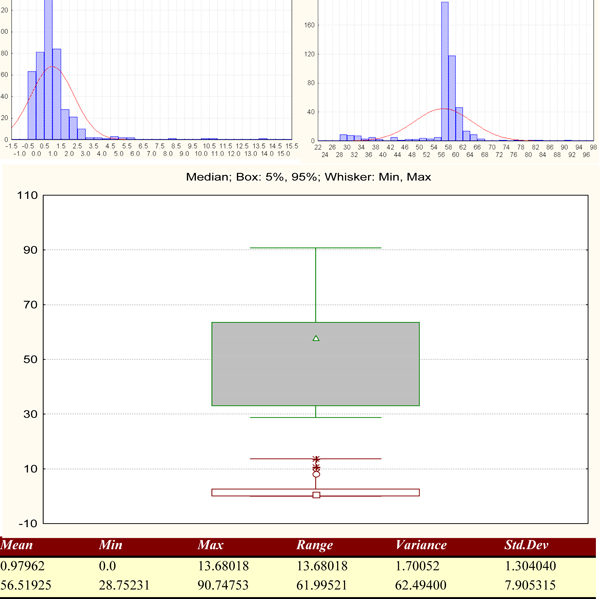
Plots and basic descriptive statistics of the local similarity function *F(W*_*i*_*) *values for matching and non-matching pairs.

To formalize this observation we have conducted a Monte-Carlo experiment. A sufficiently large set (100 000) of sequence pairs, sharing a region of local similarity was compared to a similar data set of unrelated (randomized) sequences. To make this experiment as realistic as possible the sequences were randomly chosen from the EST database. The local similarity area was introduced by coping 40 base pairs (with 2 mismatches) from a random location in a given EST sequence to a random location in its' random counterpart. The span of local similarity was chosen to represent a resolution ability twice as good as that of *d2_cluster *program (80 base pair with 95% of identity). Each pair-wise comparison of two simulated sequences can be treated as a point of *w*-dimensional space, where *w *is a number of categories of *F(W*_*i*_*) *distribution. Weight factors , required for calculation of the general similarity index are linear coefficients in the equation of the line, connecting centers of two contrasting sets (pairs with and without a local similarity). Each pair of sequences falls into one of two distinctively separate classes with nearly perfect separation (see Figure 4). A general similarity index of any pair of sequence can be seen as a projection of the categorized *F(W*_*i*_*) *distribution  to the line stretched between centroids of those simulated classes. A threshold, separating similar sequences from non-similar can be chosen so that:



This algorithm was developed specifically for EST clustering. It cannot be applied to compare protein sequences, it not sensitive enough to weak similarities between evolutionary-related sequences. High sensitivity to weak similarity is not required for clustering ESTs, where only fragmented copies of the same gene must be put in the same cluster. Excess sensitivity would also cause a problem generating false positive matches in the case of paralogous transcripts.

The implementation of our nucleotide sequence-matching algorithm uses binary encoding, similar to that of Cray Bioinformatics Library . This approach reduces the memory consumption and accelerates computation. Each word extracted from the encoded sequence serves as an index in the in the query sequence hash-table. The algorithm operates with only integer numbers, which also improves the performance. The computation is reduced to a few CPU instructions per base pair plus a short vector multiplication per whole sequence.

## Results

### Summary of the match detection algorithm

1. Prepare hash table *H *for the query sequence *S*_*q*_

2. Slide a short frame along the sequence S_*b *_by one position at a time, for each position count the number of words identical between query hash table *H *and the frame. Register all values of local similarity function in categorized form *F(W)*.

3. Calculate a single similarity score by vector multiplication of F(W) by pre-calculated weight factors .

4. Compare the resulting score to the pre-selected threshold.

### Clustering algorithm

To cluster the matching fragments we apply a single-linkage agglomerative algorithm. In the initial state each cluster has exactly one member. All initial clusters are compared to each other and matching clusters are merged until no more matches can be found.

The inter-cluster distance is chosen to be the nearest neighbor distance, i.e. the shortest distance between two objects that belong to different clusters. It has the same properties as the inter-object distance and bears all its' advantages.

**Figure 5:**
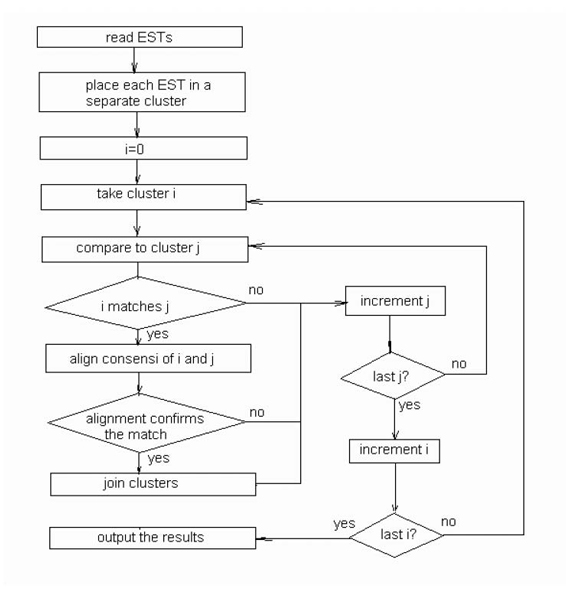
Basic algorithms of the CLU clustering program.

The process starts with a number of clusters equal to the number of initial ESTs, each cluster containing one sequence only. All clusters are arranged in a bi-directional dynamic list. All members of the list are repeatedly compared against each other. Only consensus sequences are compared in order to detect a match between clusters. Comparison is performed by application of the fast linear algorithm described above. Each time a match is found two clusters are merged, their member lists are concatenated and the consensus sequence is regenerated to make better representation for all members. The consensus sequence is produced by a pair-wise alignment procedure [11]. Each cluster in the dynamic list is compared against the rest of the list in a cycle. This cycle is repeated until no match is found for any of the clusters. The list of clusters shrinks with every cluster merge making the main cycle shorter and accelerating the clustering process. The algorithm used by the EST clustering program is schematically shown on Figure 5.

### Parameter space evaluation

**Figure 6:**
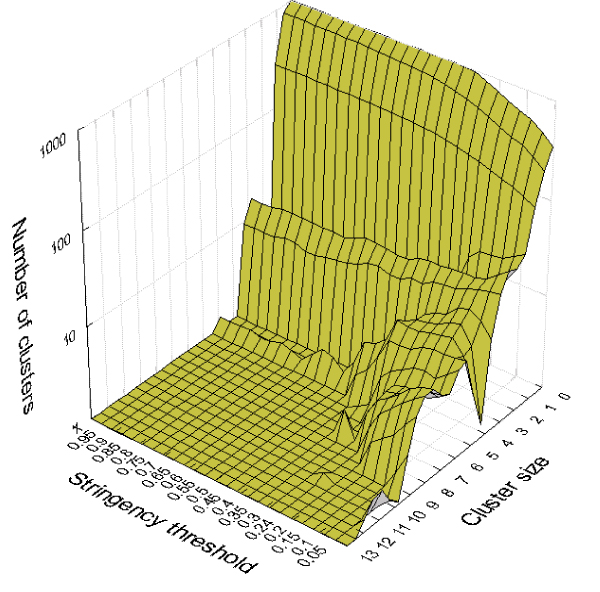
Effect of stringency threshold variation on the clustering results. The only free parameter in both loose and stringent clustering applications is the stringency threshold. This picture shows distribution of cluster size (axis X – categories by cluster size from 1 to 13, axis Z – number of clusters of certain size, decimal logarithmic scale) after clustering of 885 human EST with a threshold value (parameter) ranging from 0.05 to 0.9 (axis Y) with a step of 0.05.

Most of the parameters for clustering have been determined during development of the sequence comparison algorithm. The only parameter which remains to be set by user is the threshold value for the general similarity index. This parameter affects the stringency of clustering. A higher threshold value results in more stringent clustering with fewer and smaller clusters and higher similarity to consensus within clusters. Lower threshold results in a larger number of bigger clusters for the price of possibly less representative consensus sequence. Any specific threshold value defines a more or less arbitrary point of equilibrium between higher degree of clustering and higher cluster quality. A single parameter affects both inter-object (EST to EST within a cluster) and inter-cluster distance. When stringency threshold approaches minimum every pair of sequences produce general similarity index above exceeding the threshold. As a consequence, all initial sequences stick together in one cluster. In the opposite case, when stringency is too high even slightly dissimilar sequences produce similarity index below threshold. The result of over-stringent clustering is all singletons. The effect of stringency threshold on the number of clusters and cluster size distribution is given on Figure 6.

**Figure 7:**
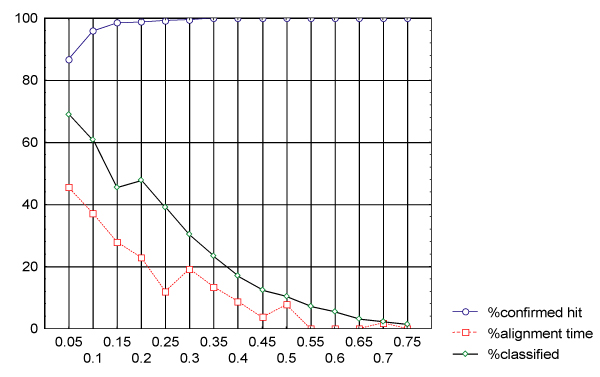
Effect of stringency threshold parameter variation. Stringency threshold affects the percentage of similarities confirmed by alignment, relative time spent on pair-wise alignment and percentage of initial ESTs assigned to clusters (non-singletons).

There are two measured statistics, which may help to optimize the stringency parameter. The stringent clustering application calculates the time spent in the alignment procedure relative to the general processing time. Under normal conditions i.e. clustering raw EST data with optimal stringency settings, this figure should be small. If the amount of time spent in the alignment procedure rises above the acceptable limit, this means that either the data set is enriched with matching sequences or that initial fast sequence comparison produces too many false-positive matches and stringency parameter has to be adjusted. Another measured statistic is percentage of initially detected matches confirmed by following thorough pair-wise alignment. This measure characterizes the rate of false-positive matches on the fast comparison stage. If ratio approaches 100% the stringency is too high and a considerable number of matches may be lost (see Figure 7). The stringency should be chosen so that percentage of confirmed matches stay low while the time, spent in alignment remains within acceptable limits.

### Estimation of clustering performance

**Figure 8:**
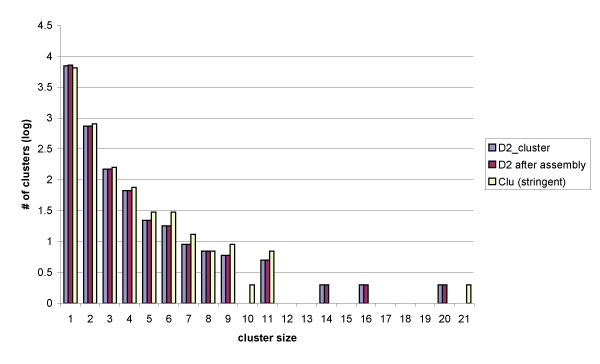
Comparison of cluster size distribution in first 10 000 human EST from eye tissue set.

Performance of the clustering programs was estimated from the analysis of the same data as has been previously used [12]. This data set contains the first 10000 ESTs from the eye tissue subset, prepared for the STACK_PACK system . Benchmark10000 (10000 ESTs from eye tissue set) is a real data set large enough to represent variations of EST sequence length, repeat and vector sequence contamination, typical data quality and redundancy. The results of testing of different programs are published on Internet and can be found at . The dataset itself can also be downloaded from the same URL. Although we don't have means to compare the quality of clustering, we suggest that as long as all tested programs produce similar results, their clustering quality is more or less the same. At least one of the tested programs, D2_cluster, is widely used and has been established as producing valid and useful results from the scientific point of view [13]. Thus, the other EST clustering systems are expected to produce a similar cluster structure. As a quick estimation of similarity of the results we propose a distribution of number of ESTs per cluster. The similarity between distributions doesn't guarantee the identity of clustering results. On the other hand, dissimilarity of distributions would be a good indicator of differences in resulting cluster structure. Our experiments show a remarkable correspondence between the result of D2_cluster and the new clustering programs, while the new programs are consistently faster. Figure 8 shows distributions of cluster size in raw D2_cluster output, D2_cluster output after cluster assembly stage and the new stringent clustering program output.

The number of singletons generated by D2_cluster is 7006, while new program generates 6531. The largest cluster defined by D2_cluster has 56 ESTs. However, after the following cluster assembly stage some of the initial EST matches are not confirmed and this cluster is split into smaller sub-clusters defined as producing a single contig. The largest cluster after assembly counts not more then 20 members. Three largest clusters produced by CLU have 66, 69 and 123 members confirmed by alignment resulting in a continuous consensus sequence. Overall CLU produces results with a very similar cluster size distribution (see Figure 8). The difference is mainly on the extreme ends of the distribution because CLU tends to produce more clusters, clusters of bigger size and leaves fewer singletons. A quick comparison of the cluster contents shows that CLU is more sensitive, especially on shorter sequences. The results of such comparison are shown in Table 1. The first 10 clusters are picked by their numbers as they appear in the D2_cluster output. In both cases of disagreement between D2_cluster and CLU results all ESTs are longer than 100 base pairs (default frame length parameter in D2_cluster), but sequences missed by D2_cluster are shorter than the others in the same cluster.

## Discussion

The CLU algorithm has been implemented as a working prototype, able to perform the most basic of the EST clustering functions – isolation of clusters, alignment and consensus generation. As a standalone application this program can be very practical for analysis of small-to-medium size EST libraries, custom microarray design, etc. The current implementation doesn't keep the alignments of clusters for analysis and generates a cluster consensus based on unsorted pair-wise alignments only. The program performs both clustering and cluster assembly, but the quality of results is curbed by the performance limitation of a desktop PC. Fast sequential pair-wise alignment implemented in the prototype version is only tolerable in a prototype-level testing, and it can't compete in accuracy with the tools, specifically developed for sequence assembly, like PHRAP. Improvement of the consensus generation and introduction of multiple alignments is the priority in development of the stringent clustering application. Sequential pair-wise alignment of low-quality EST may reduce consensus quality and is significantly affected by the order of alignments. The introduction of a more sophisticated multiple alignment will generate more representative consensus and correct this problem.

A major improvement can be an introduction of a pre-processing stage. This stage can be based on one of the fast sub-linear algorithms. Unlike the systems initially built around fast sub-linear sequence comparison, our system doesn't experience the problem of excessive false matches, is not supported by further assembly and doesn't rely on extensive pre-processing. The additional fast comparison stage should aim not to detect the matches, but rather to cut most obviously non-matching ESTs from further comparison. Development of a sub-linear booster for pre-selecting ESTs before comparison is currently in progress at Pennington Biomedical Research Center. Another improvement currently being implemented is a scalable parallel version of the CLU EST clustering algorithm for high-performance grid computing.

An implementation of the CLU algorithm can be downloaded from . The C++ code is provided free of charge. This version requires an input file in XML format. A program for converting standard FASTA format into CLU input is also provided.

## Authors' contributions

AP carried out the main load of algorithm development, prototype programming and testing as well as writing the paper. WH formulated the objectives of the project and supervised the development.

## Figures and Tables

**Figure 2:**
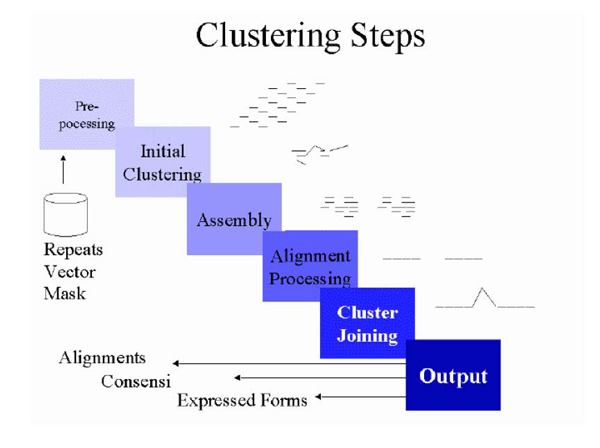
Basic EST clustering steps.

**Table 1:** Comparison of cluster contents between D2 and CLU (new clustering program) results. 10 clusters, produced by d2 from the benchmark10000 dataset with numbers from 1 to 10 are compared against corresponding CLU clusters. Due to the differences in algorithms, clusters containing the same ESTs have different numbers. In two cases of 10 (clusters #5 and #7) CLU clusters are bigger. Following alignment (available from the author upon request) confirms that additional ESTs belong to the corresponding clusters and align well.

Stack Cluster #	size	ESTs	Clu Cluster #	size	ESTs	difference
1	8	T27877 H37900 H38651 H38682 H84662 H85197 H89941 H84148	3145	8	T27877 H37900 H38651 H38682 H84662 H85197 H89941 H84148	

2	2	T27878 AA489885	7763	2	T27878 AA489885	

3	2	T27889 AA176889	5505	2	T27889 AA176889	

4	2	T27893 H84548	1040	2	T27893 H84548	

5	3	T27897 H37921 H40706	2240	4	T27897 H37921 H40706 H92170	H92170

6	6	T27899 H87764 H86519 AA057721 AA167121 AA489902	7780	6	T27899 H87764 H86519 AA057721 AA167121 AA489902	

7	2	T27904 AA063476	4532	4	T27904 AA063476 H40639 H38672	H40639 H38672

8	4	T27908 H37775 H85549 H86568	4051	5	T27908 H37775 H85549 H86568 H40669	

9	3	T27910 H80800 AA062794	4444	3	T27910 H80800 AA062794	

10	6	T27914 AA063475 AA057847 AA174102 AA219283 AA219467	6434	6	T27914 AA063475 AA057847 AA174102 AA219283 AA219467	
